# Using lot quality assurance sampling to assess access to water, sanitation and hygiene services in a refugee camp setting in South Sudan: a feasibility study

**DOI:** 10.1186/s12889-017-4656-2

**Published:** 2017-08-08

**Authors:** Elizabeth Harding, Colin Beckworth, Jean-Francois Fesselet, Annick Lenglet, Richard Lako, Joseph J. Valadez

**Affiliations:** 1 Médecins Sans Frontières, Plantage Middenlaan 14, 1018 DD Amsterdam, Netherlands; 2 Liverpool School of Tropical Medicine, Pembroke Pl, Liverpool, Merseyside, L3 5QA UK; 3 Ministry of Health, Juba, South Sudan

**Keywords:** South Sudan, Lot quality assurance sampling, LQAS, Refugee health, Humanitarian assistance, Water, Sanitation and hygiene, Monitoring and evaluation, Maban county

## Abstract

**Background:**

Humanitarian agencies working in refugee camp settings require rapid assessment methods to measure the needs of the populations they serve. Due to the high level of dependency of refugees, agencies need to carry out these assessments. Lot Quality Assurance Sampling (LQAS) is a method commonly used in development settings to assess populations living in a project catchment area to identify their greatest needs. LQAS could be well suited to serve the needs of refugee populations, but it has rarely been used in humanitarian settings. We adapted and implemented an LQAS survey design in Batil refugee camp, South Sudan in May 2013 to measure the added value of using it for sub-camp level assessment.

**Methods:**

Using pre-existing divisions within the camp, we divided the Batil catchment area into six contiguous segments, called ‘supervision areas’ (SA). Six teams of two data collectors randomly selected 19 respondents in each SA, who they interviewed to collect information on water, sanitation, hygiene, and diarrhoea prevalence. These findings were aggregated into a stratified random sample of 114 respondents, and the results were analysed to produce a coverage estimate with 95% confidence interval for the camp and to prioritize SAs within the camp.

**Results:**

The survey provided coverage estimates on WASH indicators as well as evidence that areas of the camp closer to the main road, to clinics and to the market were better served than areas at the periphery of the camp. This assumption did not hold for all services, however, as sanitation services were uniformly high regardless of location. While it was necessary to adapt the standard LQAS protocol used in low-resource communities, the LQAS model proved to be feasible in a refugee camp setting, and program managers found the results useful at both the catchment area and SA level.

**Conclusions:**

This study, one of the few adaptations of LQAS for a camp setting, shows that it is a feasible method for regular monitoring, with the added value of enabling camp managers to identify and advocate for the least served areas within the camp. Feedback on the results from stakeholders was overwhelmingly positive.

## Background

In 2000 the Sphere humanitarian charter and minimum standards in humanitarian response was first published in an attempt to establish minimum acceptable standards in humanitarian practice [1]. Since then, other codes and standards aimed at establishing accountability have followed [2]. Ensuring minimum standards are met requires measurement “across time, programmes and contexts” [3]. During the very early stages of an emergency, rapid assessment tools based on non-probability samples are appropriate [4]. However, after the immediate emergency response phase is over, cross-sectional surveys using probability sampling are recommended to guide service delivery, planning and management because early rapid epidemiological assessments can save lives [5, 6].

In the humanitarian setting, cluster surveys have been a commonly used method [7]. However, they require specialist skills to be conducted accurately, in particular for calculating the ‘design’ effect or the effect the cluster design has on statistical power [8]. Sample sizes are often large (depending on the desired survey outcome) while time and financial and human resources are frequently limited or unavailable [9, 10]. Additionally, Leaning et al. stress the ethical requirement to ensure access and equity in the allocation of resources within refugee populations, but cluster surveys only represent the average coverage across the surveyed population, since no inference can be made at the cluster level [11, 12]. A survey methodology which can make inferences both for the surveyed area and for sub-regions within the surveyed area has the potential to provide information on how equitably resources are distributed within a refugee population.

Lot Quality Assurance Sampling (LQAS) was originally developed as a classification method for industrial quality controlworks [13]. It was adapted for use in health to classify management units, referred to as ‘supervision areas’ (SA), according to coverage targets [14–16]. A sample size is set at the SA level and a decision rule selected. This is the cut-off below which the area is classified as low performance for an indicator. The decision rule depends on the sample size per SA, on the thresholds for classifying high and low performance, and on selection of two maximum tolerable misclassification errors. These are the risk of misclassifying an SA with low coverage as high (β error), and the risk of misclassifying an SA with high coverage as low (α error). Once the SAs have been classified, the samples from each SA are aggregated to calculate a coverage estimate for the entire survey catchment area [15].

LQAS offers solutions to the problems with cluster surveys posed above. Like cluster surveys, it produces a coverage estimate with a confidence interval for the survey study area. However, unlike cluster surveys, by using the decision rule as described above it is possible to examine variations in coverage within the survey study area. This gives an extra level of information to program managers, allowing them to identify inequities in service provision and to direct resources to underserved populations [17]. Organisations providing essential services within a refugee camp could potentially use LQAS to identify pockets of low coverage where the program is failing to reach the population and then strengthen service delivery teams allocated to serve specific portions of a camp. Furthermore, because it approximates a stratified random sample, there is no design effect and therefore no need to calculate one [18]. The simplicity of the method, combined with ready to use resources, has meant that the technique has been used by international organisations for more than 20 years for program monitoring and evaluation and reviewed by the World Health Organisation (WHO) as a practical method of program assessment [19–21]. However, it has rarely been used in humanitarian settings including in refugee camps [22, 23]. A global review of LQAS surveys found that LQAS had been used in post disaster settings, but none described the challenges of implementing LQAS in a refugee camp [19]. We identified only one study (unpublished) using LQAS in a camp setting in Yida camp, South Sudan [24]. Although the methodology was well described, the objective of the survey was for program monitoring and evaluation purposes and not to examine the use of LQAS in refugee camps. Moreover, little attempt was made in that study to analyse data at the SA level, a key feature of LQAS methodology.

In May 2013 we conducted an LQAS survey in Batil refugee camp, South Sudan, which at the time housed approximately 38,000 refugees from Sudan. Registration of refugees was provided by the United Nations Refugee Agency [25]. Our objectives were to document what adaptations were required to use the methodology in a camp setting and to investigate the added value of using LQAS at the sub-camp level to camp managers from the non-governmental organisation (NGO) Médecins Sans Frontières (MSF), who were providing water, sanitation and hygiene services (WASH) within the camp.

## Methods

We used a standard survey protocol widely applied in low resource settings for community health program assessment [21]. We first defined the survey catchment area as the entirety of Batil camp. We then divided the survey catchment area into discrete and contiguous SAs based on how services were provided in the camp. For this survey, we used *n* = 19 per SA with upper and lower thresholds being 30% apart. This sample size is commonly used as it provides a good balance between statistical precision and logistical efficiency [13, 15, 17].

**Fig. 1:**
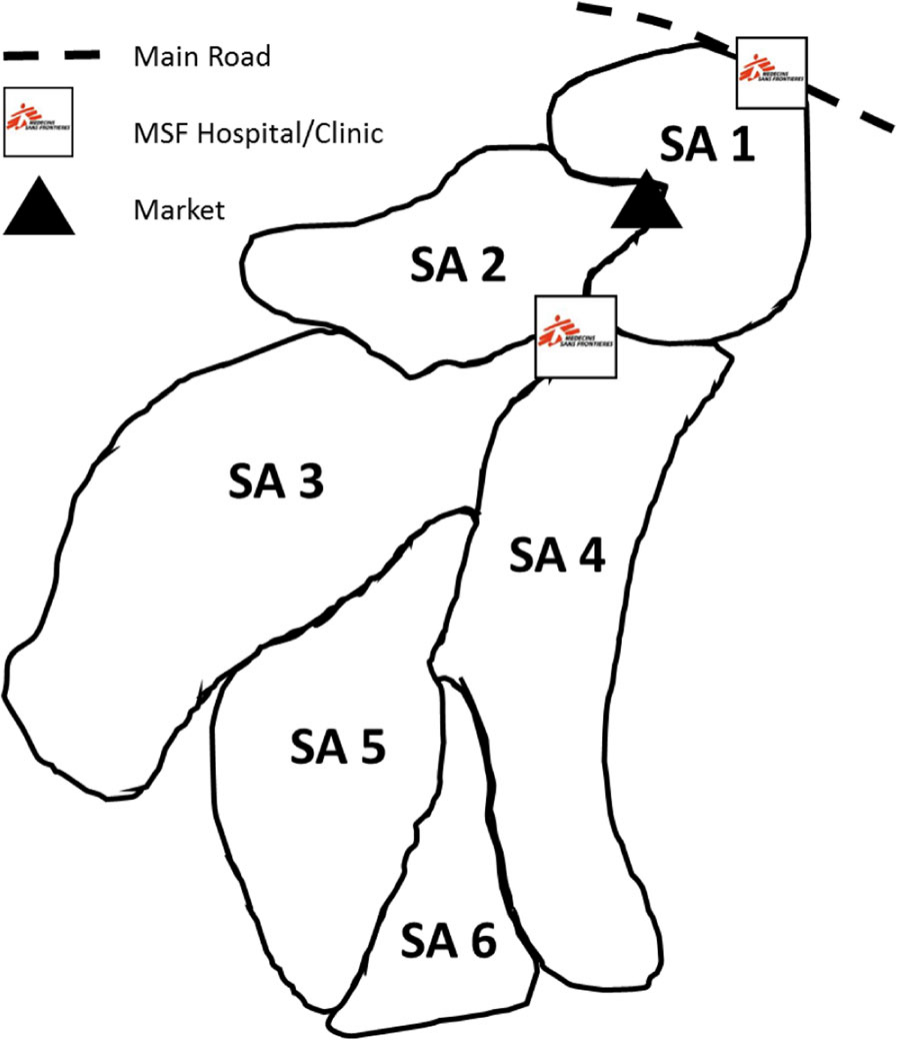
Map of Batil camp with six Supervision Areas

Adapting the SA to the context of a refugee camp was challenging, since there were none of the obvious geographic demarcations that are found in more formal settlements. We consulted local program managers from MSF and found that previous to our study, the camp had been divided into 12 approximately equal segments to facilitate outreach work. For logistical reasons, 12 SAs was seen as impractical for this setting as it resulted in a sample size of 19 × 12 = 228. Therefore, together with local camp managers, we combined the 12 outreach areas to form six SAs (Fig. 1). This resulted in an overall sample size of 19 × 6 = 114. This decision was deemed acceptable to camp managers both in terms of logistical efforts and the statistical precision of information gained at the SA and survey catchment area level. Because the aim of the survey was to provide data specific to measuring MSF WASH programs in the camp, consultation was limited to managers of the MSF WASH programs.

Indicators for the survey were selected to report on the outcomes of the MSF WASH program. Wherever possible, indicators and attendant question sets were taken from existing sources using accepted definitions. Examples include using an improved water source and an improved sanitation facility. The source for these indicators was UNICEFs Multiple Indicator Cluster Survey [26]. However, LQAS is principally a tool for monitoring and evaluating programs, and the purpose of the survey was to evaluate the reach and effectiveness of the MSF WASH program. Therefore, it was also necessary to design certain indicators which were specific to the outcomes of the program. For example, households which had been visited by a health promoter within the last 2 weeks, and households which owned an Ibrik (a water storage container used for washing). These indicators were designed in accordance with best practices as described by Markiewicz and Patrick [27].

Another important adaptation of the LQAS methodology was the random process used to select respondents within each SA. Usually, the sampling frame consists of a complete list of formal settlements or communities (frequently a village) in an SA. Probability proportional to size (PPS) sampling is then used to randomly allocate the 19 interview locations. But in Batil camp refugees did not have formal communities, so we had to search for alternative ways to divide the SAs. Local program managers were aware of pre-existing divisions, which demarcated areas of responsibility for local leaders, called Sheik villages. Each SA in Batil camp consisted of between 8 and 12 Sheik villages. We used the Sheik villages in place of formal settlements, and used PPS sampling to select Sheik villages in each SA for the 19 interviews, an approach which required knowing the population size for each Sheik village. In the case of Batil camp this information was available from the United Nations High Commission for Refugees (UNHCR). Where a Sheik village was sampled for more than one interview, each interview location was sampled randomly.

In each Sheik village, data collectors randomly selected a household using segmentation sampling [28]. Again, we had to adapt the methodology; in cases where data collectors found insufficient landmarks to use segmentation sampling, they were instructed to use the random walk technique to identify the household within the Sheik village [29, 30].

Once households were selected, the interview teams targeted two categories of respondents: heads of household and caregivers of children 0–59 months. Heads of household were asked questions relating to access to WASH services, while the caregivers of children were asked a different set of questions on whether the child had diarrhoea in the 2 weeks preceding the survey (see Table 1 for a list of indicators used in the survey). If one or both categories of respondents were present in the same household then they were interviewed. If either of the respondents was not present, the data collector moved to the next nearest door until one respondent from each group was found. When both types of respondent were interviewed, the data collector moved on to the next randomly selected interview location. This parallel sampling technique resulted in two discrete samples of 19 respondents in each SA at little extra effort (one of heads of household, the other of caregivers of children 0–59 months). As the information gathered from each respondent group was analysed independently, they were treated in aggregate as stratified random samples rather than as cluster samples.

**Table 1:** LQAS results for six supervision areas in Batil camp

Indicator	SA1	SA2	SA3	SA4	SA5	SA6	Weighted Average % (DR)	95% Confidence Interval	Target (DR)
Water									
Proportion of households using an improved water source	19	19	19	16	19	19	97.3% (NA)	±2.8%	95% (16)
Proportion of households who report that water was continuously available from their habitual water source for the last 2 weeks	16	9^b^	8^a, b^	9^b^	14	8^a, b^	57.0% (9)	±8.6%	80% (13)
Proportion of households which own an Ibrik^c^	16	4^a, b^	3^a, b^	10	10	8^b^	45.2% (7)	±8.0%	60% (9)
Hygiene									
Proportion of households with at least one piece of soap (observed)	18	9^b^	2^a, b^	13	8^b^	6^a, b^	51.1% (8)	±7.7%	75% (12)
Proportion of households with a hand washing area within the living area	18	5^b^	0^a, b^	0^a, b^	1^a, b^	5^b^	27.2% (3)	±5.3%	60% (9)
Proportion of households which have been visited by a health promoter within the last 2 weeks	19	18	19	19	15^a, b^	16	93.5% (16)	±4.1%	95% (16)
Proportion of heads of households who know four critical moments to practice hand washing with soap	18	7^b^	1^a, b^	11^b^	0^a, b^	2^a, b^	37.0% (5)	±6.4%	75% (12)
Proportion of households who have a hand washing area with soap and water adjacent to their habitual sanitation facility	9	9	1^a, b^	1^a, b^	3^b^	8^b^	27.9% (3)	±7.6%	60% (9)
Sanitation									
Proportion of households who usually use an improved sanitation facility	19	18	18	19	19	17	96.8% (NA)	±3.1%	
Proportion of households that do not practice open defecation	19	18	18	19	19	17	96.8% (NA)	±3.1%	
Diarrhea prevalence									
Prevalence of diarrhoea among children 0–59 months in the last 2 weeks	4	5	7	6	7	3	28.2%	±8.2%	

^a^SA has not met the decision rule for average coverage for the camp

^b^SA has not met the decision rule for the target set by program managers

^d^An Ibrik is a water storage container used for washing

We adapted standard LQAS training materials used for community surveys for data collector training in this refugee camp setting [21]. Adaptations included reducing the days of training from four to three so we could concentrate on only those skills needed for effective data collection: random sampling and using the questionnaire. Preparing the sampling strategy and recruiting and training the 12 data collectors took 9 days; data collection took 4 days; hand tabulation and analysis took 3 days. After a total of 16 days in country, we could make the results available to programme managers.

### Data analyses

Firstly, we aggregated the data for each respondent group from the six SAs as a stratified random sample to calculate a weighted coverage estimate for Batil camp for key indicators. The weights were the population sizes of each SA. Secondly, we classified each SA as a high or low priority. In this initial application of LQAS in a refugee camp setting, we worked closely with program managers to set a target for each indicator (for example, 75% percent of households should have at least one piece of soap) and selected a corresponding LQAS decision rule to classify each SA as having met or not met its target [21]. Thirdly, we used average coverage within the camp for each indicator to again classify SAs. This approach uses LQAS as a test for homogeneity to identify outlier SAs which are well below average and therefore priorities for action [21]. All classifications of the indicators were made by hand tabulating the raw data. The prevalence with 95% confidence intervals (CI) was calculated using an Excel spreadsheet. We applied these low-tech approaches because in a refugee camp setting specialist software is often not available. Analyses were completed within 2 days of the data collection.

To further evaluate the use of LQAS in a camp setting, we carried out two additional studies. Firstly, we conducted key informant interviews of MSF program managers on their opinion of the challenges and added value of the methodology. Secondly, we performed a sensitivity analysis of different analytic approaches, comparing prevalence measures produced by using: crude unweighted data; data weighted by the SA population; and calculating prevalence using the survey design, including a finite population correction using Stata V12.

### Ethical aspects

The survey was carried out by the Liverpool School of Tropical Medicine (LSTM) in collaboration with MSF together with the Ministry of Health of the Republic of South Sudan. As the survey addressed an important aspect of monitoring and evaluation for WASH activities, it fulfilled the exemption criteria for Ethical Review Board approval from MSF. It was also approved by the MSF Medical Director, by the Ministry of Health for South Sudan, and by the Liverpool School of Tropical Medicine’s ethics review committee.

## Results

### Catchment area level results

Our results revealed that 97.3% (95% CI 94.5–100%) of camp respondents had access to water from an improved source, but in the 2 weeks preceding the survey, water supply had been uninterupted for 57.0% (95% CI 48.4–65.6%) of respondents (see Table 1). Results for hygiene indicators revealed that 45.2% (95% CI 37.2–53.2%) of all households possessed their own Ibrik, and 51.1% (95% CI 43.4–58.8%) could produce at least one piece of soap when asked. However, only 27.2% (95% CI 21.9–32.5%) of households and 27.9% (95% CI 20.3–35.5%) of sanitation facilities had a designated place for hand washing where water and soap were immediately available, and only 37.0% (95% CI 30.6–43.4%) of respondents could state four critical moments to wash hands. Nevertheless, 96.8% (95% CI 93.7–99.9%) of respondents habitually used an improved sanitation facility and only 3.2% (95% CI 0.1–6.3%) practiced defecation in an open area. A total of 28.2% (95% CI 20.0–36.4%) of caregivers of children 0–59 months reported their child had suffered from diarrhoea in the two-weeks preceding the survey.

### Supervision area level results

Results at the SA level revealed that the average condition of the camp masked important variations. At the camp periphery, SA-3 and SA-6 were classified as the priority SA as they lacked consistent access to water from an improved source. For hygiene indicators, SA-3 was again the area of highest priority, having been classified in the low category for both program and average coverage targets for four out of six hygiene indicators. However, for the sanitation indicators, no SA was in the low category, meaning that for sanitation, there was only a small amount of variation among the SAs, with overall coverage being high. SA-1 was the best performer, being in the high category for all indicators. It was also located closest to amenities such as clinics, the main road and the market. It was not classified in the low category for any indicator either as a target or as an average.

In summary, water and hygiene indicators tended to be lower the further respondents were from the main camp amenities. This effect held for households reporting that their water supply was interrupted in the last two-weeks; for those not having a handwashing point within the living area; and for those not owning an Ibrik. However, this association was not absolute since SA-5 was further from the amenities than SA-3, and this association did not hold for sanitation indicators, which were uniformly high across the camp.

### Key informant interviews

Feedback from the key informant interviews was overwhelmingly positive. Participants felt that the division of the camp into six SAs was a useful way to identify priority areas within the camp, even though these six areas were amalgamations of 12 pre-existing outreach areas. Participants also noted that because LQAS generated data in a standardized way, it would be easier to compare results across time and programs, and to advocate with other organisations providing services in the camp. All stakeholders observed that LQAS was a useful tool for identifying priorities within the camp for baseline assessment and ongoing monitoring.

The challenges mentioned included: programme managers required guidance to define SAs so that they provided results which were useful for guiding service provision; programme managers were unfamiliar with the LQAS technique and had to be informed as to what inferences could and could not be made at the SA and catchment area level. Finding staff with the requisite skills to carry out the survey was also a challenge. Data collectors had to be recruited from the refugee populations so that they had the requisite language skills; feedback from the data collectors suggested that many of the concepts and much of the language associated with random sampling were unfamiliar. Finally, applying the techniques of random sampling to select households was found to be difficult in some areas; boundaries between Sheik villages sometimes overlapped and the Sheik had to be relied on to identify which tents belonged to which Sheik village.

### Sensitivity analysis

Table 2 presents three sets of results: 1) crude unweighted proportions; 2) proportions weighted by the SA population size; and 3) proportions calculated with Stata V12 using the survey design. The largest difference in coverage estimates was −0.027 between the survey design and the crude proportion; the average difference in coverage when comparing all permutations of the three forms of analysis was 0.008 with the crude proportion having the smaller average estimates. The largest difference in standard error (SE) was 0.014 with the crude proportion having the largest value; however, the largest mean effect for the difference in proportions was SE = 0.005. The largest difference in the range of the 95% confidence interval was 0.0278 with the crude proportion having the largest range; the largest average difference in confidence interval was 0.01 for the crude proportion. The differences produced by these approaches were minimal.

**Table 2:** Sensitivity analysis of coverage using crude, weighted and the survey design in the calculations

Indicators	Crude Coverage			Weighted Coverage			Crude - Weighted			Coverage with Survey Design			Crude - Survey Design			(Crude - Weighted) - (Crude - Survey Design)		
	Coverage	Standard Error	95% CI	Coverage	Standard Error	95% CI	Coverage Difference	SE Difference	CI Difference	Coverage	Standard Error	95% CI	Coverage Difference	SE Difference	CI Difference	Difference in Difference in Coverage	Difference in Difference in SE	Difference in Difference in CI
Water																		
Proportion of households using an improved water source	0.973	0.015	0.030	0.973	0.014	0.028	0.000	0.001	0.002	0.973	0.015	0.029	0.000	0.001	0.001	0.000	0.000	0.001
Proportion of households who report that water was continuously available from their habitual water source for the last 2 weeks	0.561	0.047	0.092	0.570	0.044	0.087	-0.009	0.003	0.005	0.570	0.045	0.089	-0.009	0.002	0.003	0.000	0.001	0.002
Proportion of households which own an Ibrik†	0.451	0.047	0.093	0.455	0.041	0.082	-0.004	0.006	0.011	0.455	0.042	0.084	-0.004	0.005	0.009	0.000	0.001	0.002
Hygiene																		
Proportion of households with at least one piece of soap (observed)	0.487	0.047	0.094	0.506	0.040	0.079	-0.020	0.008	0.015	0.508	0.041	0.080	-0.021	0.007	0.013	0.002	0.001	0.002
Proportion of households with a hand washing area within the living area	0.257	0.041	0.082	0.274	0.027	0.054	-0.017	0.014	0.028	0.274	0.028	0.055	-0.017	0.014	0.027	0.000	0.000	0.001
Proportion of households which have been visited by a health promoter within the last 2 weeks	0.938	0.023	0.045	0.941	0.021	0.041	-0.003	0.002	0.004	0.942	0.021	0.041	-0.004	0.002	0.004	0.000	0.000	0.001
Proportion of heads of households who know four critical moments to practice hand washing with soap	0.345	0.045	0.089	0.370	0.033	0.065	-0.025	0.012	0.024	0.372	0.034	0.067	-0.027	0.011	0.022	0.002	0.001	0.002
Sanitation																		
Proportion of households who usually use an improved sanitation facility	0.965	0.017	0.035	0.967	0.016	0.032	-0.003	0.001	0.003	0.968	0.016	0.032	-0.003	0.001	0.002	0.001	0.000	0.000
Proportion of households that practice open defecation	0.035	0.017	0.035	0.033	0.016	0.032	0.003	0.001	0.003	0.032	0.016	0.032	0.003	0.001	0.002	-0.001	0.000	0.000
Diarrhoea																		
Prevalence of diarrhoea among children 0-59 months in the last 2 weeks	0.283	0.043	0.084	0.283	0.042	0.084	0.000	0.000	0.001	0.284	0.043	0.086	-0.001	-0.001	-0.002	0.001	0.001	0.002
Standard Deviation of Difference							0.010	0.005	0.010				0.010	0.005	0.010	0.001	0.000	0.001
Maximum Difference							0.003	0.014	0.028				0.003	0.014	0.027	0.002	0.001	0.002
Average Difference							-0.008	0.005	0.010				-0.008	0.004	0.008	0.000	0.001	0.001
Median Difference							-0.004	0.002	0.005				-0.004	0.002	0.004	0.000	0.001	0.001

^†^An ibrik is a water storage container used for washing

## Discussion

This paper is the first peer-reviewed study assessing an adaptation of LQAS for a camp setting. The survey provided coverage estimates on WASH indicators as well as providing evidence that SA-3 in the camp was underperforming on six of the eight indicators and thus was the priority area for immediate intervention. Adaptations to the standard LQAS protocol used in low resource communities was required, but we have shown that it is a feasible undertaking, and one that program managers found useful at both the catchment area and SA level.

Most of the adaptations we made were in selecting the sample. Community LQAS uses existing administrative divisions and population data to form the sampling frame for SAs. The study area lacked formal administrative divisions so we relied on informal divisions identified by camp managers. Whilst these were meaningful to the MSF WASH program managers, we were not able to assess how readily useable this way of presenting information was to other NGOs providing services (including others providing WASH services) in the camp using other service delivery schemes. A guiding principle for the United Nations Office for the Coordination of Humanitarian Affairs (OCHA) is accessibility of information [31]. Other authors have written on the detrimental effect of incompatibility of information in humanitarian information exchange [32]. However, feedback from the program managers stated that in their opinion, the LQAS information would be useful for advocacy with other organisations.

Despite the informal nature of the division of the camp, we were able to identify population data for each division, supplied by the UNHCR. However, in newly displaced groups, populations may not be organised into coherent geographic areas and population data required for weighting results may not always be available [6]. In this case, spatial mapping of refugee populations using methods such as those described by Boccardo and Tonolo and methods to estimate population density such as those described by Checchi et al. could be used, although as the authors explain, there are limitations to these methodologies [33, 34]. As our sensitivity analysis shows, there was at most a − 0.027 difference in weighted and unweighted results, although on average the difference was just −0.008, with crude coverage having the smaller estimates. The largest difference in SE was 0.014 with the crude proportion having the largest SE; the mean effect on SE = 0.005 for the crude proportion. The largest difference in the range of the 95% confidence interval was 0.0278 with the crude proportion having the largest range; the average difference in confidence interval was 0.01 for the crude proportion. These differences are small and not meaningful for program management. Therefore, we conclude that for rapid assessment in a refugee context the crude proportion can be used by program managers if they can accept a small reduction in precision.

Another adaptation was the use of the random walk method to select households where segmentation sampling proved infeasible due to lack of landmarks. Some authors have claimed that this method biases household selection toward the point from where the walk begins [28]. Other authors found that whilst segmentation sampling is preferred, the random walk method is an acceptable alternative when applied correctly [35]. By restricting use of the random walk to small segments of the camp with few houses (or tents) in our survey, we think that bias was probably minimal.

In summary, whilst the use of a stratified random sample design posed challenges, these were all overcome and the data was collected in 4 days by 12 data collectors working in teams of two. The confidence interval for this sample size did not exceed ±0.094. Whilst a cluster sample design is resource efficient, it generally requires a large sample size to achieve the same confidence interval because of its design effect, that is, the increased variance in the sample resulting from the cluster sample design. Although design effect is calculated after the survey is complete, an assumption of a design effect of 1.5 is commonly used, meaning that the sample size for a comparable cluster sample would be 1.5 times larger than that of a stratified random sample [8, 36]. Whilst the most accurate estimate of the proportions for the indicators are those calculated using the survey design, we doubt that the slight variation for the crude values is epidemiologically meaningful. Rather than burden humanitarian workers with this level of precision, the crude proportion is most likely adequate.

There was unanimous feedback from program managers that the added value of LQAS was to unmask the existing variations in different SAs without amplifying the sample size. We identified that areas of the camp closer to the main road, to MSF clinics and to the market were better served than areas at the periphery of the camp, with the result that program managers could redirect resources to areas of greatest need. Whilst the conclusion that areas furthest from the centre of the camp are not as well served may seem self-evident, it must be remembered that the purpose of LQAS is to identify the “worst of the worst” served areas. Our survey area had three SAs which could be described as far from the centre of the camp; identifying which one of the three peripheral areas was least served was seen by program managers as valuable. Also, the assumption that all peripheral areas would be worst served did not hold for all services; sanitation services, for example, were uniformly high regardless of location. Program managers found that LQAS was sufficiently easy to use to be managed by field staff but still provided rigorous, statistically defensible results which could be used to communicate with coordination bodies. Examples of organisations with which to share information are UNHCR, who were responsible for coordination in Batil Camp, or OCHA, whose remit is to “mobilize and coordinate effective and principled humanitarian action” [37]. Finally, information might also be used to advocate for greater inputs to least-served areas from other NGOs working within a camp.

We also found that programme managers who were unfamiliar with the LQAS technique required guidance on what inferences could and could not be made at the SA and catchment area level and in setting decision rules. We used a well-tested approach to implementing LQAS [21]. Other authors have suggested an alternative approach leading to more SAs being classified as a priority [38]. Such survey designs emphasize identifying the “*best of the best*” versus our intent which was to identify the “*worst of the worst*” SAs. While it is beyond the scope of this paper to debate this issue, which has been adequately done elsewhere, our experience of working in humanitarian settings tells us it is essential to use scarce resources to resolve service delivery problems in the most pernicious locations [17]. Our proposed design does this and is appropriate for a refugee camp. Our aim was to identify SAs which were the worst performing with the largest proportion of people at risk to health problems. MSF could then direct its scarce materials and human resources to these areas, an approach also favoured by other field-based organisations [39].

Alternative approaches to setting decision rules have also been described in the literature. Alberti et al. described an LQAS survey to evaluate the coverage of an emergency measles vaccination campaign in Chad [40]. In this survey, SA sample sizes were calculated according to error terms set by the authors, resulting in a sample size of 65 respondents per SA. By comparison, our survey used a smaller SA sample of 19. The difference in precision between these two samples sizes is found in the difference between the upper and the lower threshold, described above. In our study, the upper and lower thresholds were 30 percentage points apart; in the Alberti study the upper and lower thresholds were 15 percentage points apart. The justification for this in the Alberti study was that the target coverage was 85%, and the authors thought it important to identify all SAs where vaccination coverage fell below 70%. Our objective was to identify the best and worst performing SAs, therefore a 30% difference between thresholds was deemed acceptable. The decision rules and error terms when *n* = 19 are readily available, meaning that no calculations were required and the smaller sample size lessened the impact on already stretched logistics [15, 21]. However, using LQAS means that users can adapt the design to suit their own needs. The statistical tables already exist to support these adaptations [15].

In this paper, we have not disaggregated results at the supervision area level by gender. The *n* = 19 used in each SA of Batil camp is sufficient for classification of the SA with alpha and beta errors that do not exceed 0.10. However, if we stratify the *n* = 19 we lose statistical precision. If we assume *n* = 10 for the same pU and pL then alpha and beta increase to 0.12 and 0.17 respectively. In this case, providing sex disaggregated data may in fact be misleading due the increased probability of an error. Also, the indicators we measured are household level data making the gender stratification perhaps not essential. Were we to want gender stratification at the SA level in the future then we would increase the sample size accordingly. However, gender stratification at the camp level would be possible and render about the same precision as a cluster sample that did the same. While our not stratifying the data at the SA level is a limitation of our current design it still reveals insights into camp functioning at a high level of granularity not provided by other approaches.

## Conclusion

Our study demonstrates that standard community based LQAS is feasible in a camp setting. The survey provided an estimate for water and sanitation access for camp residents, much the same as a standard cluster sample survey. The added value of LQAS was that it enabled camp managers (and the MSF project) to quickly identify and advocate for the least-served areas within this camp in terms of water and sanitation. The limitations of the study were that the UNHCR population data used for the sampling frame is not always available in camp settings. Next steps are to investigate whether LQAS can be used in camps where no population data is available, and for monitoring projects within a camp in a number of different settings. It seems evident that we also need to test the use of LQAS during the acute phase of an emergency.

## Abbreviations

CI: Confidence interval
LQAS: Lot quality assurance sampling
LSTM: Liverpool School of Tropical Medicine
MSF: Médecins Sans Frontières
NGO: Non-governmental organisation
OCHA: Organisation for the coordination of humanitarian assistance
PPS: Probability proportional to size
SA: Supervision area
SE: Standard error
UNHCR: The UN Refugee Agency
WASH: Water, sanitation and hygiene
WHO: World Health Organisation
